# Retraction: Transcultural validation of the “revised sport motivation scale” (SMS‐II) in Arabic language: Exploratory study on motivation in sport for a sample of Tunisian Athletes

**DOI:** 10.1371/journal.pone.0318595

**Published:** 2025-01-29

**Authors:** The PLOS One Editors

The *PLOS One* Editors retract this article [1] due to concerns about compliance with the PLOS Human Subjects Research policy, the integrity of the underlying data, and the reliability of the published results. Additional concerns were raised about the authorship of the article. The authors’ responses did not resolve these concerns. PLOS regrets that the issues were not identified prior to the article’s publication.

MB did not agree with the retraction. AA, AT, MSK, MAM, and ABA either did not respond directly or could not be reached.
